# A Radiosensitivity Prediction Model Developed Based on Weighted Correlation Network Analysis of Hypoxia Genes for Lower-Grade Glioma

**DOI:** 10.3389/fonc.2022.757686

**Published:** 2022-02-25

**Authors:** Zixuan Du, Hanshan Liu, Lu Bai, Derui Yan, Huijun Li, Sun Peng, JianPing Cao, Song-Bai Liu, Zaixiang Tang

**Affiliations:** ^1^ Department of Biostatistics and Jiangsu Key Laboratory of Preventive and Translational Medicine for Geriatric Diseases, School of Public Health, Medical College of Soochow University, Suzhou, China; ^2^ Suzhou Key Laboratory of Medical Biotechnology, Suzhou Vocational Health College, Suzhou, China; ^3^ Department of Medical Oncology, Jiangsu Provincial Corps Hospital, Chinese People’s Armed Police Forces, Yangzhou City, China; ^4^ Department of Otolaryngology, The First Affiliated Hospital of Soochow University, Suzhou, China; ^5^ School of Radiation Medicine and Protection and Collaborative Innovation Center of Radiation Medicine of Jiangsu Higher Education Institutions, Soochow University, Suzhou, China

**Keywords:** lower-grade glioma, radiosensitivity prediction model, radiosensitivity, Lasso, WGCNA

## Abstract

**Background and Purpose:**

Hypoxia is one of the basic characteristics of the physical microenvironment of solid tumors. The relationship between radiotherapy and hypoxia is complex. However, there is no radiosensitivity prediction model based on hypoxia genes. We attempted to construct a radiosensitivity prediction model developed based on hypoxia genes for lower-grade glioma (LGG) by using weighted correlation network analysis (WGCNA) and least absolute shrinkage and selection operator (Lasso).

**Methods:**

In this research, radiotherapy-related module genes were selected after WGCNA. Then, Lasso was performed to select genes in patients who received radiotherapy. Finally, 12 genes (*AGK*, *ETV4*, *PARD6A*, *PTP4A2*, *RIOK3*, *SIGMAR1*, *SLC34A2*, *SMURF1*, *STK33*, *TCEAL1*, *TFPI*, and *UROS*) were included in the model. A radiosensitivity-related risk score model was established based on the overall rate of The Cancer Genome Atlas (TCGA) dataset in patients who received radiotherapy. The model was validated in TCGA dataset and two Chinese Glioma Genome Atlas (CGGA) datasets. A novel nomogram was developed to predict the overall survival of LGG patients.

**Results:**

We developed and verified a radiosensitivity-related risk score model based on hypoxia genes. The radiosensitivity-related risk score served as an independent prognostic indicator. This radiosensitivity-related risk score model has prognostic prediction ability. Moreover, a nomogram integrating risk score with age and tumor grade was established to perform better for predicting 1-, 3-, and 5-year survival rates.

**Conclusions:**

We developed and validated a radiosensitivity prediction model that can be used by clinicians and researchers to predict patient survival rates and achieve personalized treatment of LGG.

## Introduction

Lower-grade glioma (LGG) consists of diffuse low-grade and intermediate-grade gliomas (World Health Organization grades II and III) (1). For the first time, in the WHO 2016 classification of gliomas, gliomas were defined based on the presence/absence of isocitrate dehydrogenase (IDH) mutation and 1p/19q codeletion (2). This is a transition from histological classification to molecular classification, and it provides strong support for the individualized treatment of LGG patients.

Treatments for LGG usually include surgery, chemotherapy, immunotherapy, and radiotherapy. One study showed that radiotherapy can increase progression-free survival (PFS) and improve the overall survival (OS) of LGG patients (3). A nationwide analysis of LGG patients found that radiotherapy was associated with improved survival outcomes (4). However, due to individual differences, some patients showed radiation toxicity after receiving radiotherapy. The radiosensitivity of tumors is the key factor in determining the curative effect of radiotherapy. The purpose of predicting the radiosensitivity of patients is to identify the population sensitive to radiotherapy and maximize the treatment benefit of radiotherapy. Thus, it is imperative to exploit new treatments closely related to radiotherapy for LGG to improve the prognosis.

The occurrence and development of tumors are related to the excessive proliferation and reduced apoptosis of tumor cells. The hypoxic microenvironment promoted the growth, infiltration, and metastasis of tumor cells. Hypoxia is one of the basic characteristics of the physical microenvironment of solid tumors and can influence immune cell functions (5). Tumor hypoxia and the resulting energy metabolism of tumor cells are important features of cancer and also the driving force and basis of cancer metastasis. Hypoxic conditions are considered to be a feasible approach for targeted immunotherapy (6).

The relationship between radiotherapy and hypoxia is complex. Radiotherapy is the targeted administration of X-rays to destroy cancer cells and tumor tissue. It targets rapidly proliferating tumor cells by inducing oxidative stress through increased reactive oxygen species (ROS) (7). Hypoxia condition is the main factor of tumor radiation resistance (8). Tumor cells in hypoxic conditions thus attain aggressive phenotypes and become resistant to chemo- and radiotherapies resulting in higher mortality (9). In addition to the well-known protective effect of hypoxia on the radiological responses of cells and tissues, hypoxic conditions can also lead to altered gene expression patterns, resulting in more or less genomic alterations in different cell populations (10).

Lin et al. developed a hypoxia signature to evaluate and predict prognosis in glioma, and this model reflected overall immune response intensity in the glioma microenvironment (11). Wang et al. developed a risk signature with five genes that could serve as an independent factor for predicting the prognosis of patients with glioblastoma (GBM) (12). Xiao et al. explored a three-gene signature as a candidate prognostic biomarker for LGG (13). Likewise, Li et al. developed a radiosensitive gene signature by using coexpression and ceRNA network analysis to select genes (14). However, a model for predicting the benefit of radiotherapy based on hypoxia-related genes by using weighted correlation network analysis (WGCNA) in LGGs has not been established.

In this study, WGCNA was used to screen the most relevant radiotherapy module. This study aims to develop a radiotherapy signature related to hypoxia-related genes to provide survival and radiotherapy response prediction for LGG patients.

## Materials and Methods

### Data Sources

**Figure 1:**
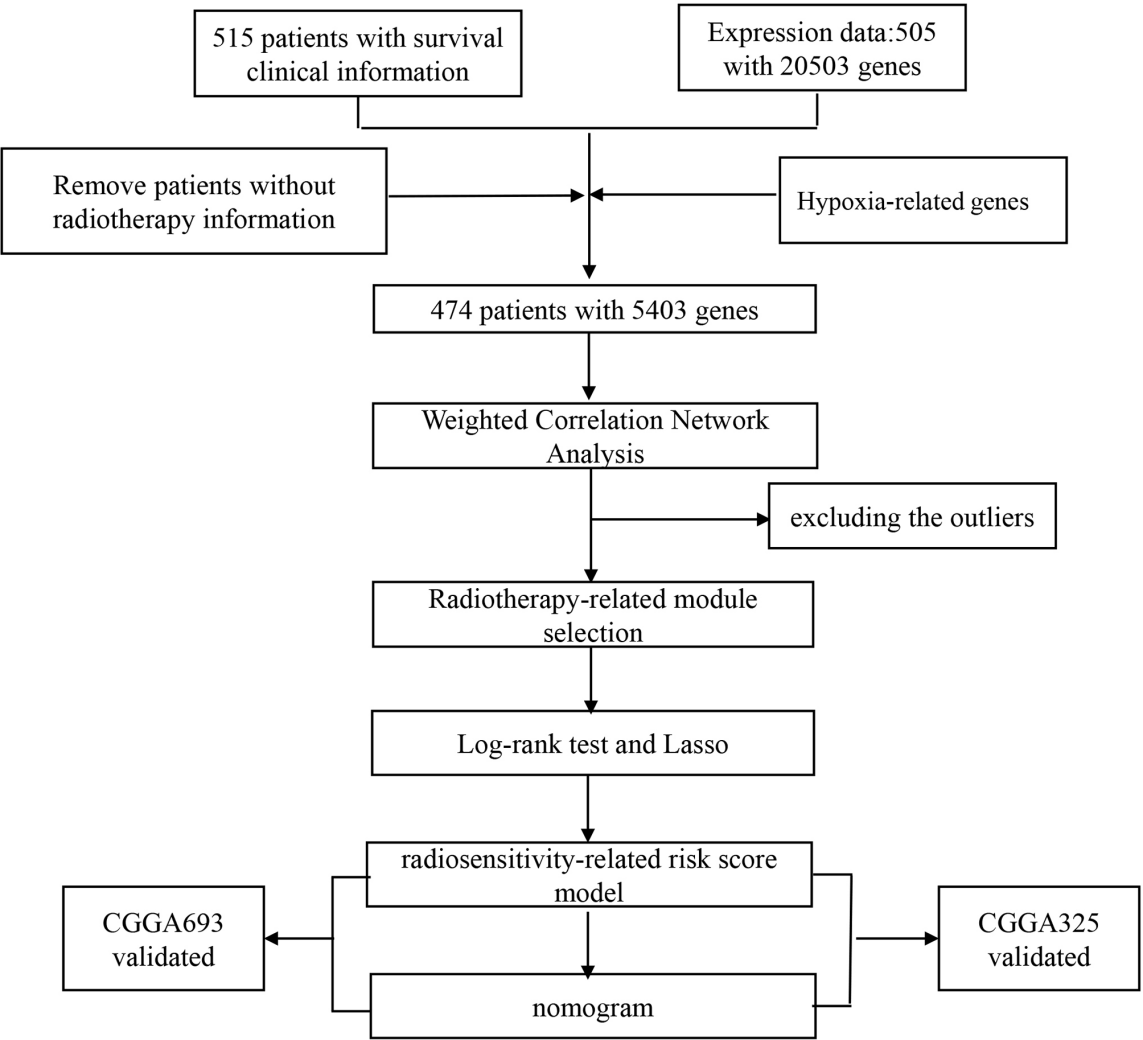
The flowchart of study design, patient selection, and gene selection.

LGG patients with clinical and gene expression files were downloaded from a public database The Cancer Genome Atlas (TCGA; http://cancergenome.nih.gov/) by using the R package TCGA-Assembler (15). LGG patients with survival information were procured from the UCSC Cancer Genomics Browser (https://xenabrowser.net/datapages/) (16). We used OS and PFS as endpoints and removed those without radiotherapy information (n = 29) and survival information (n = 3). Hypoxia-related genes were extracted from GeneCards (https://www.genecards.org/). Genes with a common symbol name in TCGA were selected. The flowchart is summarized in **Figure 1**. Finally, we obtained 466 patients with 5,403 hypoxia-related genes for analysis. Gene expression and clinical profiles of 443 LGG patients (CGGA693 dataset) (17, 18) and 182 LGG patients (CGGA325 dataset) (19, 20) were downloaded as external validation datasets from the Chinese Glioma Genome Atlas (CGGA) dataset (http://www.cgga.org.cn/). The RNA-seq transcriptome data were estimated as log2(x + 1) transformed. The cleaned clinical data are summarized in **Supplementary Tables 1**, **2**.

### Weighted Correlation Network Analysis

WGCNA can identify highly related genes in thousands of genes and cluster them into modules and then was used to establish the relationship between phenotypic traits and gene expression data. By calculating the correlation degree between the gene module and the external clinicopathological information, we can obtain the module genes highly related to the clinicopathological information and obtain the hub genes. WGCNA can be implemented by R package WGCNA (21).

In our study, WGCNA was performed to discover radiotherapy-related genes. We analyzed hypoxia-related genes and clinical data, including OS status, PFS status, age, grade, radiotherapy, and treatment response. First, hierarchical clustering analysis was utilized to exclude the outliers. Subsequently, the “pickSoftThreshold” function was performed to estimate the value of the powers. The R-squared criterion was set to 0.9. Pearson’s correlation matrices were used for all pairs of genes, and the weighted adjacency matrix was constructed using the power function. After the power was selected, the adjacency matrix was converted to a topological overlap matrix (TOM). Genes with similar expression profiles were classified into gene modules, and hierarchical clustering was performed by the class average method based on TOM. The minimum gene size in each module was set as 30. To further analyze the modules, the dissimilarity of module eigengenes was calculated, and some modules were merged. The merged cutoff threshold was set to 0.2, which meant that modules with a similarity higher than 0.8 were merged into one module. Then, the correlations between modules and clinical factors of LGG were investigated by using Pearson’s correlation test. Finally, the genes of the most significant radiotherapy-related module were chosen for subsequent analysis.

### Functional Enrichment Analysis

To obtain the function of genes in the radiotherapy-related module, we performed the Gene Ontology (GO) and Kyoto Encyclopedia of Genes and Genomes (KEGG) analyses by using the R clusterprofiler (22). The GO analysis included biological processes (BPs), cellular component (CC), and molecular function (MF).

### Definition of Radiosensitivity and Radiosensitivity Prediction Model

In our study, radiosensitivity for the patients was defined in terms of survival benefit (**Supplementary Figure 1**). 1) In patients who received radiotherapy, patients in group A had a better survival rate than the patients in group B. Then patients in group A could be defined as radiosensitive patients (RS group). 2) In patients who did not receive radiotherapy, the survival rate of group A (RS group) was not better (equal or worse) than that of the other group.

The radiosensitivity prediction model was constructed in the patients who had received radiotherapy. Patients who received radiotherapy provided more information related to radiosensitivity. We defined a radiosensitivity prediction model for the patients satisfying both of the following criteria: the constructed radiation sensitivity correlation model constructed can be used to divide the population into a high-risk group and a low-risk group. 1) In the radiotherapy patients, the survival rate of the low-risk group was higher than that of the high-risk group. 2) There was no significant difference in survival between the high- and low-risk groups in the group that did not receive radiation therapy. The low-risk group was defined as the RS group, and the high-risk group was defined as the radioresistant (RR) group. Therefore, the radiosensitivity model can select the patients with better benefit from radiotherapy, while the results in the population without radiotherapy can better show that the model is related to radiosensitivity.

### Radiosensitivity-Related Risk Score Construction

A log-rank test was applied to assess the relationship between the expression of genes in radiotherapy-related modules and the OS of radiotherapy patients in TCGA. Whole LGG patients were divided into the high- and low-expression level groups using the median gene expression level as a cutoff point. The RNAs with log−rank p < 0.05 in the radiotherapy patients and log-rank p > 0.05 in the non-radiotherapy patients were identified as radiosensitivity−related RNAs. Then, the least absolute shrinkage and selection operator (Lasso) regression was performed to narrow the range of genes in patients who received radiotherapy. The radiosensitivity-related risk score was computed as follows:

Radiosensitivity−related risk score = Σ(βRNAn × exprRNAn)

### Radiosensitivity-Related Risk Score Validation

The LGG patients were divided into the high- and low-risk groups with the median radiosensitivity-related risk score as the cutoff. The Kaplan–Meier method was used to plot survival curves. Time-dependent receiver operating characteristic (ROC) curve analysis was used to evaluate the prognostic value. The radiosensitivity-related risk score was validated in TCGA and two CGGA datasets.

### The Radiosensitivity-Related Risk Score Is an Independent Prognostic Indicator

Univariate and multivariate Cox proportional hazard regression analyses were used to examine whether the radiosensitivity-related risk score was an independent prognostic factor. The forest plot was plotted to show the hazard ratio (HR) and 95% CIs.

### Development and Validation of the Nomogram

To evaluate the 1-, 3-, and 5-year survival probability for patients with LGG, a nomogram model including all independent prognostic factors was built for LGG patients in TCGA. The nomogram model was validated with the PFS of TCGA and two CGGA datasets.

### Analysis Method

All statistical analyses were performed using R software (4.0.2). WGCNA was performed by using the “WGCNA” R package. Lasso analysis was conducted by using the “glmnet” R package. A bilateral p-value <0.05 was considered statistically significant.

## Results

### Weighted Coexpression Network Construction and Identification of Radiotherapy-Related Modules

**Figure 2:**
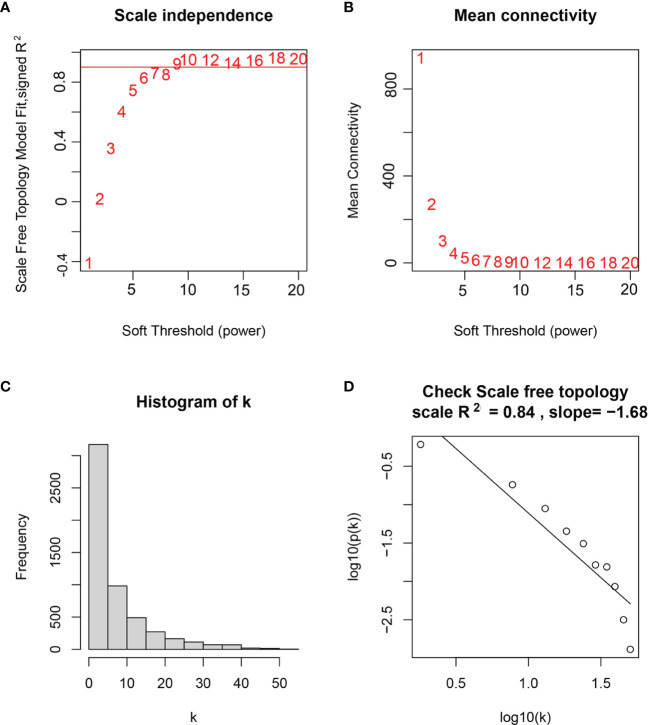
Selection of weighted value β. **(A)** Determine the weighted value β that satisfies the law of scale-free networks. **(B)** Determine the soft threshold based on the network connectivity. **(C)** β = 7, the connection degree of each node in the network histogram distribution. **(D)** The scale-free topology test.

**Figure 3:**
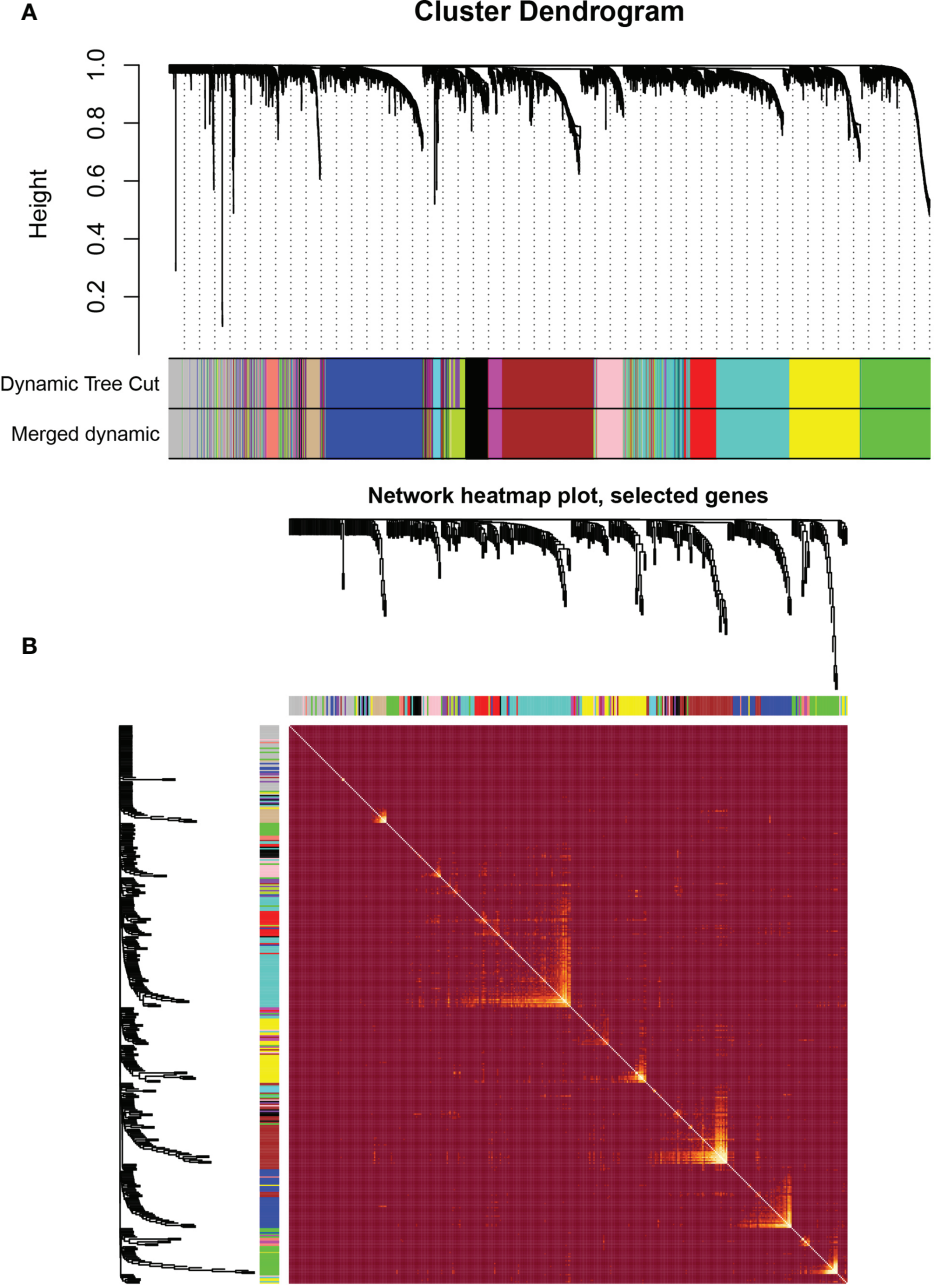
Weighted correlation network analysis. **(A)** Clustering dendrogram of genes based on topological overlapping. **(B)** Network heatmap of the whole genes.

**Figure 4:**
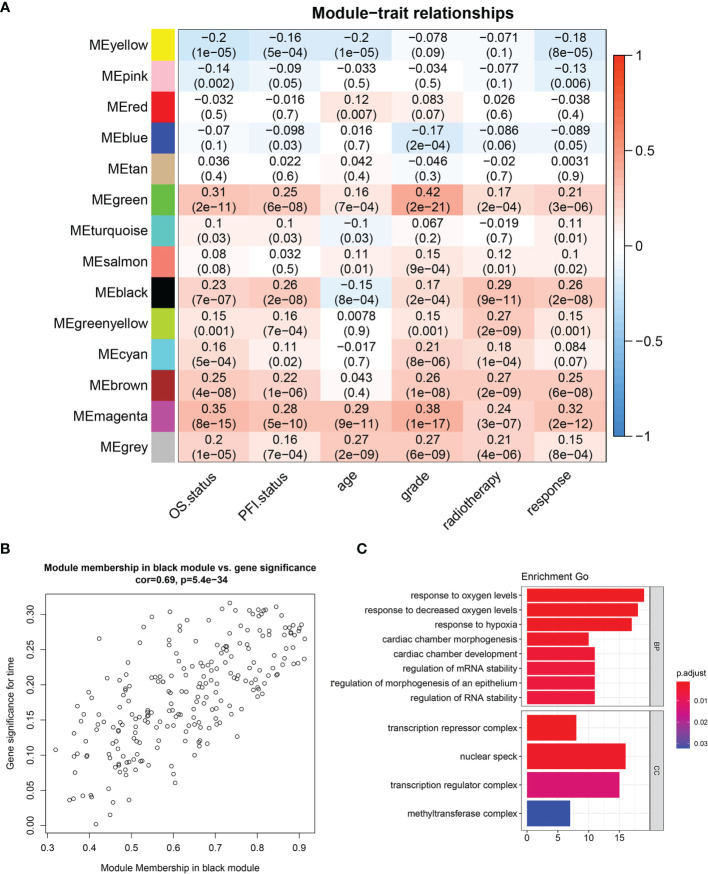
Identification of significant modules. **(A)** Module trait relationship heatmap. **(B)** Scatterplot of gene significance for radiotherapy (y-axis) vs. module membership (x-axis) in the black module. **(C)** GO enrichment analysis of genes in radiotherapy module. BP, biological process; MF, molecular function; CC, cellular component; GO, Gene Ontology.

WGCNA was performed in TCGA-LGG dataset to determine the coexpression network most highly associated with the radiotherapy modules. The hclust function was used to determine if there were any outliers (**Supplementary Figure 2**). A total of 466 samples were in the clusters after removing 8 outliers in the samples based on the average linkage method. When the soft threshold power value was β = 7 and the scale R^2^ = 0.84, the average connectivity of the RNA group was high, and the connectivity between genes conformed to the scale-free network distribution (**Figures 2A, B** **)**. The scale-free topological fitting index R-square was calculated to reach 0.84 (**Figures 2C, D**). Next, the TOM was constructed (**Figure 3A**), and a topological overlapping heatmap was depicted of the TOM including the top 400 genes (**Figure 3B**). A total of 13 modules were identified from the RNA coexpression network after merging modules with a similarity higher than 0.8. The relationships between gene modules and clinical traits are shown in a heatmap (**Figure 4A**). Thus, the black module was considered to have the highest correlation with radiotherapy (r = 0.69, p < 0.001) and was considered a “radiotherapy-related module” (**Figure 4B**).

### Functional Analysis of Genes Radiotherapy-Related Module

GO analysis was performed to analyze the function of the radiotherapy-related module (**Figure 4C**). We discovered that the radiotherapy-related module was functionally associated with responding to oxygen levels, responding to decreased oxygen levels, and responding to hypoxia, cardiac chamber morphogenesis, cardiac chamber development, regulation of mRNA stability, and regulation of RNA stability. CCs include the transcription repressor complex, nuclear speck, transcription regulation complex, and methyltransferase complex.

### Construction of Radiosensitivity-Related Signature

**Figure 5:**
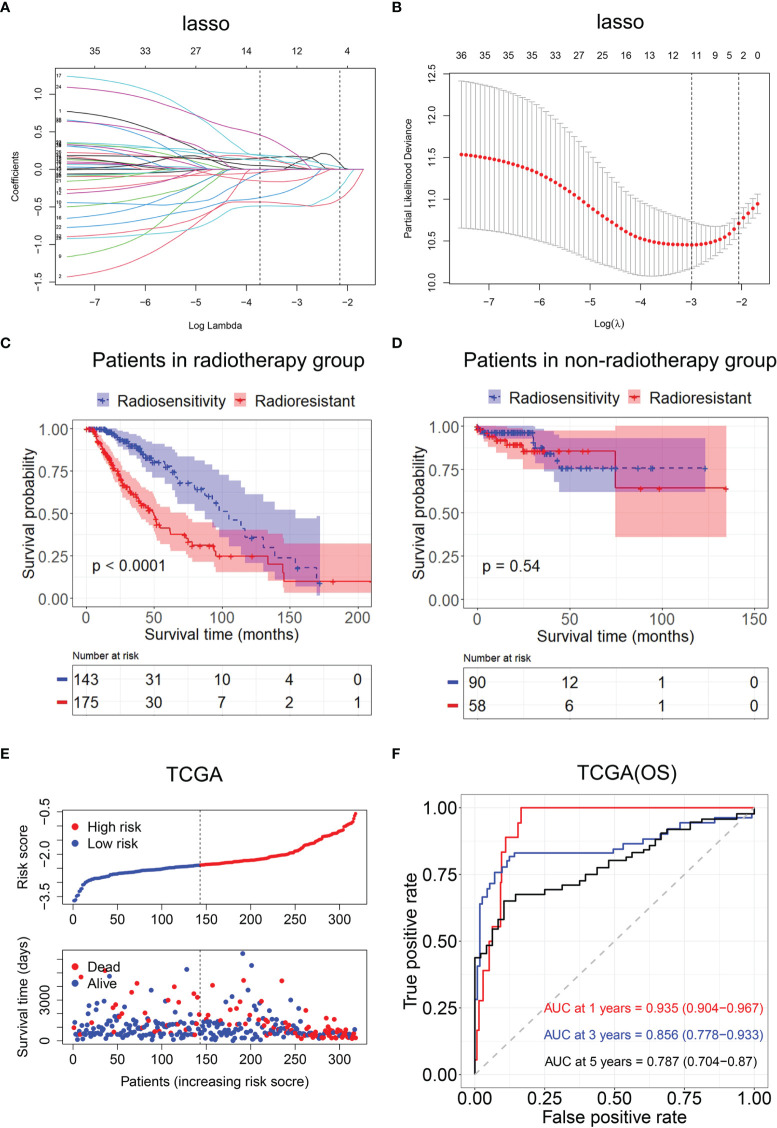
Construction of the radiosensitivity-related risk score model. **(A)** The solution paths of the Lasso. **(B)** The partial log-likelihood profiles of the Lasso. **(C)** Kaplan–Meier curves for the RS group and RR group in patients with radiotherapy and patients who did not receive radiotherapy. RR, radioresistant group; RS, radiosensitive group. **(D)** Kaplan–Meier curves for the RS group and RR group in patients who did not receive radiotherapy. RR, radioresistant group; RS, radiosensitive group. **(E)** Risk score distribution of each patient in TCGA (OS). **(F)** Time-dependent ROC curve analysis of the radiosensitivity-related risk score in TCGA (OS). OS, overall survival; TCGA, The Cancer Genome Atlas; ROC, receiver operating characteristic.

We selected modules related to radiotherapy for further analysis. A total of 231 genes were subjected to the log-rank test in radiotherapy patients and non-radiotherapy patients. Thirty-six radiosensitivity−related genes were identified in the univariate analysis. Subsequently, the Lasso Cox regression model was used to identify the most robust markers for prognosis (**Figures 5A, B** **)**. Finally, 12 genes (*AGK*, *ETV4*, *PARD6A*, *PTP4A2*, *RIOK3*, *SIGMAR1*, *SLC34A2*, *SMURF1*, *STK33*, *TCEAL1*, *TFPI*, and *UROS*) were included in the model. The radiosensitivity-related risk scores were calculated based on the linear combination of the expression levels of genes multiplied by the corresponding Lasso coefficients. The radiosensitivity-related risk score was as follows: radiosensitivity-related risk score = 0.16864 * *AGK* + 0.14242 * *ETV4* − 0.14386 * *PARD6A* + 0.00584 * *PTP4A2* − 0.09746 * *RIOK3* − 0.23212 * *SIGMAR1* + 0.07849 * *SLC34A2* + 0.18813 * *SMURF1* + 0.04491 * *STK33* − 0.4922 * *TCEAL1* + 0.01536 * *TFPI* − 0.4694 * *UROS*.

Then, the patients with LGG in TCGA dataset were divided into the high-risk (n = 233) or low-risk groups (n = 233) according to the median risk score. The Kaplan–Meier analysis revealed that OS time was significantly increased in the low-risk group compared with the high-risk group in patients who received radiotherapy (p < 0.001, **Figure 5C**). There was no difference in OS between the high-risk group and low-risk group in patients who did not receive radiotherapy (p = 0.54, **Figure 5D**). The low-risk group was defined as an RS group, and the high-risk group was defined as an RR group. The risk score distribution of each patient in TCGA is shown in **Figure 5E**.

Then, ROC analysis was used to evaluate the predictive efficiency of the radiosensitivity-related risk score model in the 1-, 3-, and 5-year survival rates (1-year area under the curve (AUC): 0.935 (0.904–0.967); 3-year AUC: 0.856 (0.778–0.933); 5-year AUC: 0.787 (0.704–0.87), **Figure 5F**).

### Validation of Radiosensitivity Model in Validation Sets

**Figure 6:**
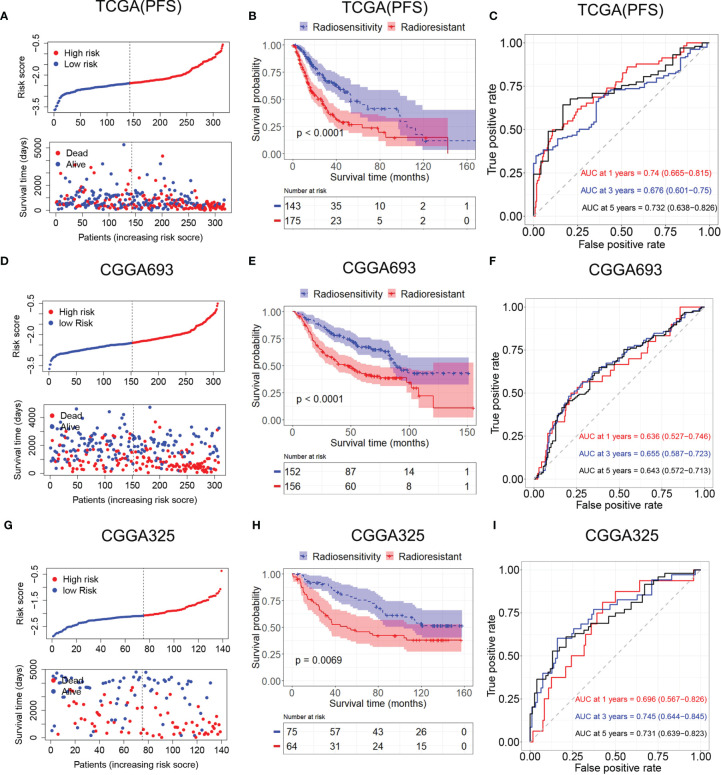
Validation of the radiosensitivity-related risk score model. **(A)** Risk score distribution of each patient in TCGA (PFS). **(B)** Kaplan–Meier curves for the RS group and RR group in patients with radiotherapy from TCGA (PFS). **(C)** Time-dependent ROC curve analysis of the radiosensitivity-related risk score in TCGA (PFS). **(D)** Risk score distribution of each patient in the CGGA693. **(E)** Kaplan–Meier curves for the RS group and RR group in patients with radiotherapy from CGGA693. **(F)** Time-dependent ROC curve analysis of the radiosensitivity-related risk score in the CGGA693. **(G)** Risk score distribution of each patient in the CGGA325. **(H)** Kaplan–Meier curves for the RS group and RR group in patients with radiotherapy from CGGA325. **(I)** Time-dependent ROC curve analysis of the radiosensitivity-related risk score in the CGGA325. TCGA, The Cancer Genome Atlas; PFS, progression-free survival; RS, radiosensitive; RR, radioresistant; ROC, receiver operating characteristic.

A radiosensitivity model was validated in TCGA with PFS as the endpoint. The Kaplan–Meier plots indicated that patients in the RR group exhibited worse PFS than patients in the RS group in patients who received radiotherapy (p < 0.001, **Figure 6B**). Time-dependent ROC analysis results showed that the AUCs of the radiosensitivity model were 0.74, 0.676, and 0.732 at survival times of 1, 3, and 5 years, respectively (**Figure 6C**). Plots of risk score distribution are shown in **Figures 6A, D, G**.

Patients in the CGGA693 and CGGA325 datasets were divided into the RS group and RR group based on the median risk score in each dataset. The Kaplan–Meier analysis showed that patients in the RS group had a more favorable outcome than patients in the RR group in patients who received radiotherapy (CGGA693, p < 0.001; CGGA325, p < 0.001; **Figures 6E, H** **)**. These results indicated the accuracy of the radiosensitivity-related signature in predicting the outcomes of LGG patients. ROC curves were used to evaluate the predictive accuracy for 1-, 3-, and 5-year survival. AUC values revealed the high predictive value of the radiosensitivity-related risk score for LGG patients (CGGA693: 1-year AUC: 0.636 (0.527–0.746); 3-year AUC: 0.655 (0.587–0.732); 5-year AUC: 0.643 (0.572–0.713); CGGA325: 1-year AUC: 0.696 (0.567–0.862); 3-year AUC: 0.745 (0.644–0.845); 5-year AUC: 0.731 (0.639–0.823), **Figures 6F, I**).

### The Radiosensitivity-Related Risk Score Is an Independent Prognostic Factor

**Figure 7:**
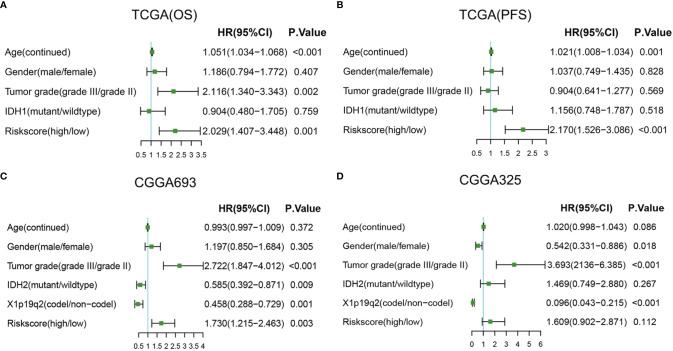
Forest plots of multivariate Cox regression. **(A)** Forest plots of multivariate Cox regression in TCGA (OS). **(B)** Forest plots of multivariate Cox regression in TCGA (PFS). **(C)** Forest plots of multivariate Cox regression in CGGA693. **(D)** Forest plots of multivariate Cox regression in CGGA325. A, astrocytoma; OA, oligoastrocytoma; O, oligodendroglioma; TCGA, The Cancer Genome Atlas; OS, overall survival; PFS, progression-free survival.

Then, univariate and multivariable Cox regression analyses were conducted to evaluate whether the radiosensitivity-related risk score is an independent prognostic factor for LGG. The results indicated that factors such as risk score and grade were significantly correlated with patient survival in both TCGA dataset and two CGGA datasets. Age (HR: 1.054, 95% CI: 1.038–1.071, p < 0.001), tumor grade (HR: 2.715, 95% CI: 1.736–4.247, p < 0.001), and risk score (HR: 2.712, 95% CI: 1.763–4.171, p < 0.001) were significantly associated with OS. The univariate analysis indicated that a high-risk score was significantly correlated with poor OS. The multivariate Cox regression results showed that the radiosensitivity-related risk score was an independent prognostic factor for LGG patients after adjusting for clinical factors such as age, sex, tumor grade, race, and IDH1. When OS was used as an endpoint, the HR was 2.029 (95% CI: 1.407–3.448, p < 0.001, **Figure 7A**). When PFS was used as an endpoint, the HR was 2.170 (HR: 2.170, 95% CI: 1.526–3.086, p < 0.001, **Figure 7B**). In CGGA datasets, we adjusted for clinical factors such as age, sex, tumor grade, race, IDH2, and X1p19q2, and the multivariate Cox regression results also demonstrated that the radiosensitivity-related risk score was an independent prognostic factor for LGG (CGGA693: HR: 1.730, 95% CI: 1.215–2.463, p = 0.003; **Figure 7C**). Unfortunately, the multivariate Cox regression result was not significant in the CGGA325 dataset (CGGA325: HR: 1.609, 95% CI: 0.902–2.871, p = 0.112; **Figure 7D**). We consider that there are too few patients in the CGGA325 database.

### Construction and Validation of Nomogram

**Figure 8:**
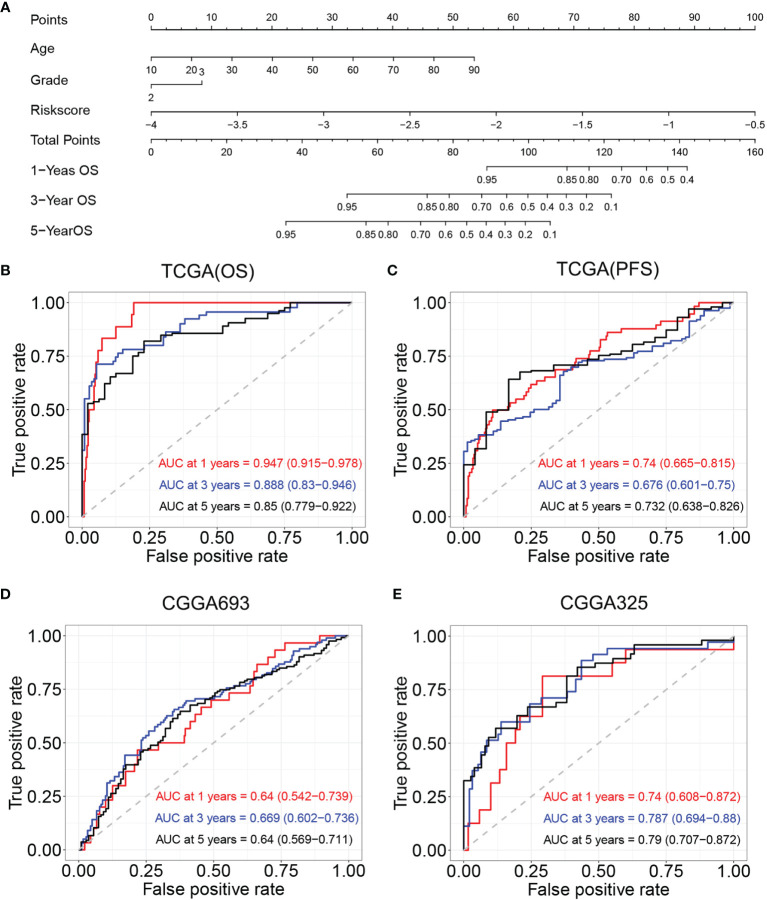
Construction and validation of nomogram model. **(A)** Nomogram model for predicting the probability of 1-, 3-, and 5-year OS in LGGs. **(B)** Time-dependent ROC curve analyses of the nomogram model in TCGA (OS). **(C)** Time-dependent ROC curve analyses of the nomogram model in TCGA (PFS). **(D)** Time-dependent ROC curve analyses of the nomogram model in the CGGA693. **(E)** Time-dependent ROC curve analyses of the nomogram model in the CGGA325. OS, overall survival; LGGs, lower-grade gliomas; ROC, receiver operating characteristic; TCGA, The Cancer Genome Atlas; PFS, progression-free survival.

Age, tumor grade, and risk score were listed as candidate indicators for nomogram construction. Then, an optimal nomogram was established combining age, tumor grade, and risk score to predict a certain clinical outcome (**Figure 8A**). **Figure 8B** shows that AUCs of the nomogram for 1-, 3-, and 5-year OS were 0.947 (0.915–0.978), 0.888 (0.83–0.946), and 0.850 (0.779–0.922), respectively, which were better than those of the models with a single risk score model. **Figure 8C** demonstrates that AUCs of the nomogram for 1-, 3-, and 5-year PFS were 0.74 (0.665–0.815), 0.676 (0.601–0.750), and 0.732 (0.638–0.826), respectively. We also used two CGGA datasets to verify a nomogram model. **Figure 8D** demonstrates that the AUCs of nomogram at 1, 3, and 5 years were 0.64 (95% CI: 0.542–0.739), 0.669 (95% CI: 0.602–0.736), and 0.64 (95% CI: 0.569–0.711), respectively, for CGGA693. **Figure 8E** demonstrates that the AUCs of nomogram at 1, 3, and 5 years were 0.740 (95% CI: 0.608–0.872), 0.787 (95% CI: 0.694–0.88), and 0.79 (95% CI: 0.707–0.872), respectively, for CGGA325. The nomogram model for the prognostic model displayed superior predictive performance as compared with the risk score in the CGGA325.

## Discussion

LGG is one of the leading causes of cancer-related death worldwide. The treatments of LGG include surgery, chemotherapy, and radiotherapy. Radiotherapy may not be appropriate for all patients due to its toxicity. Thus, it is important to develop risk scores based on genetic and clinical characteristics to help determine which patients would benefit the most from radiation therapy.

Hypoxia is a marker of the tumor microenvironment and plays an important role in tumor occurrence, development, metastasis, and metabolism (23). The relationship between hypoxia and radiotherapy is complex. ROS are essential for destroying tumor cells by ionizing radiation. Under hypoxia, oxygen reduction interferes with ROS produced by ionizing radiation, and tumor cells have developed various mechanisms for evading apoptosis mediated by HIF-1 (24). Tumor hypoxia is a serious problem for radiotherapy because radiosensitivity is gradually limited when partial oxygen pressure in the tumor is low (25).

In this study, 6,327 hypoxia-related genes were downloaded from the GeneCards website. We identified genes of the radiotherapy-related model by WGCNA. Further log-rank tests and Lasso Cox regression analyses were performed to identify 12 genes in patients who received radiotherapy. A radiosensitivity-related risk score model was established based on the OS of TCGA dataset in patients who received radiotherapy. Then, this model was validated based on the PFS of TCGA dataset and two CGGA datasets. This radiosensitivity-related risk score model has prognostic prediction ability and is an independent prognostic indicator in LGG.

Of the 12 model genes, *PARD6A*, *RIOK3*, *SIGMAR1*, *TCEAL1*, and *UROS* expression levels were positively correlated with favorable outcomes, whereas *AGK*, *ETV4*, *PTP4A2*, *SLC34A2*, *SMURF1*, *STK33*, *RCN1*, *SPP1*, *RPN2*, and *ATP2A2* expression levels were associated with adverse outcomes. *AGK* (acylglycerol kinase) is a lipid kinase. The *AGK*-*PTEN* axis is a key pathway that coordinates the glycolysis and the function of CD8+ T cells (26). There have been many research findings that *AGK* is overexpressed in many cancers, such as gastric cancer (27) and cervical squamous cell cancer (28). In glioma, the expression level of *AGK* was identified as an independent prognostic factor and associated with the poor prognosis (29). *ETV4* (ETS Translocation Variant 4) is one of an *ETS* family transcription factor and is aberrantly expressed in a variety of human tumors such as prostate cancer (30) and non-small cell lung cancer (31). *ETV4* plays a wide role in the regulation of hypoxic genes (32). The *RAS-RAF*-*MEK*-*ERK* (MAPK) signaling pathway and *PI3K*/*Akt* signaling can activate *ETV4* expression in cancer (33). *PTP4A2* (protein tyrosine phosphatase 4A2) is associated with the overall and disease-free survival of breast cancer (34). Du et al. found that high *PTP4A2* expression is associated with ROS-induced cell death, which may contribute to cancer patient survival and response to radiotherapy (35). *RIOK3* expression is increased during hypoxic exposure and increases cell migration and invasion in cancer (36). High *RIOK3* levels in gliomas contribute to proliferation, migration, and invasion of glioma cells (37). *SLC34A2* (solute carrier family 34 member A2) is a member of the *SLC34* family and is usually overexpressed in glioma tissues and cell lines. *SLC34A2* knockdown exhibited suppressive effects on cell proliferation and migration/invasion (38). *SMURF1* is involved in the regulation of cellular processes, including autophagy, growth, and cell migration. Chang et al. proved that *SMURF1* was associated with glioma cell migration (39). *STK33* (serine/threonine kinase 33) is a serine/threonine kinase and plays an important role in cancer cell proliferation (40). *TFPI* (tissue factor pathway inhibitor) has been associated with radiosensitivity or radiosensitivity in previous studies (41). However, we were unable to find a report about the relationship between LGG and *PARD6A* (partitioning defective 6 homolog alpha), *SIGMAR1* (sigma non-opioid intracellular receptor 1), *TCEAL1* (transcription elongation factor A-like 1) and *UROS* (uroporphyrinogen synthase) genes.

A novel nomogram model integrating risk score with age and tumor grade was developed to predict the OS of LGG patients. We also validated the nomogram model in two CGGA datasets. According to the radiosensitivity-related risk score and nomogram, clinicians can be able to identify a group of patients who can benefit better from radiotherapy and then can predict the 1-, 3-, and 5-year OS of LGG. Nomograms could provide probabilistic predictions for individual patients. In our study, we constructed a nomogram that can predict the OS in LGG patients. The survival rates in CGGA datasets indicate that the nomogram had a good predictive performance. At the same time, the nomogram model that integrated risk score with age and tumor grade had better predictive performance than the model constructed by a radiosensitivity-related risk score factor.

Much work thus far has focused on the relationship between hypoxia and radiotherapy in tumors. Hypoxia is an important characteristic of the tumor microenvironment, and it is closely related to the occurrence and development of tumors. Several hypoxia genes have been used to develop gene expression signatures for evaluating tumor prognosis. Liu et al. identified low hypoxia status and high immune status as factors for gastric cancer patients’ OS and developed a hypoxia-immune-based gene signature (42). A hypoxia risk model was developed in TCGA and validated in CGGA to reflect overall immune response intensity in the glioma microenvironment (11). The hypoxia-related signature was also developed and validated in breast cancer (43) and lung adenocarcinoma (44). To our knowledge, this is the first study to construct a model to predict radiotherapy sensitivity from hypoxia genes from the perspective of radiotherapy.

Our study provides new insights into the individualized treatment for LGG. The main strength of this study is that the WGCNA method was used to construct a radiosensitivity-related model in LGG patients. WGCNA can use all genes to identify the gene set of interest, and it can be associated with the sample phenotype. At the same time, WGCNA can also be applied to small samples (21). Considering the importance of hypoxia genes in the tumor microenvironment, we selected hypoxia genes to be included in the study. We focused on radiotherapy, so we used WGCNA to select the gene module most related to radiotherapy. Then Lasso Cox was used to select genes. Finally, a radiosensitivity-related model based on hypoxia genes was developed in TCGA dataset and validated in CGGA datasets. The radiosensitivity-related model can identify LGG patients most likely to benefit from radiotherapy. However, a limitation of our study is that this was a retrospective study, and the models should be further confirmed by prospective studies. From the perspective of clinical treatment, the risk score we constructed can select the people who benefit from radiotherapy, so as to improve the effect of radiotherapy. On the other hand, we can develop a test kit according to the risk score for clinical application.

In conclusion, the radiosensitivity-related score was demonstrated to be an independent prognostic factor for LGG patients. Patients with LGG can be divided into the RS and RR groups by radiosensitivity-related score. The patients in the RS group were more likely to benefit from radiotherapy. This model can be used by clinicians and researchers to predict patient survival rates and achieve personalized treatment of LGG.

## Data Availability Statement

Publicly available datasets were analyzed in this study. We obtained the patients with LGG datasets from TCGA (http://cancergenome.nih.gov/) and CGGA (http://www.cgga.org.cn/).

## Author Contributions

Study conception and design: ZD, HSL, SL, and ZT. Data collection and cleaning: ZD, DY, LB, and SL. Real data analysis and interpretation: ZD, HJL, SP, SL, and JC. Drafting of the manuscript: ZD, HSL, HJL, DY, JC, and ZT. All authors listed have made a substantial, direct, and intellectual contribution to the work and approved it for publication.

## Funding

This work was supported in part by the National Natural Science Foundation of China (81773541), funded by the Priority Academic Program Development of Jiangsu Higher Education Institutions at Soochow University, the State Key Laboratory of Radiation Medicine and Protection (GZK1201919) to ZT, National Natural Science Foundation of China (81872552, U1967220) to JC. This work was also supported by the Jiangsu higher education institution innovative research team for science and technology (2021), Key technology program of Suzhou people’s livelihood technology projects (Grant No. SKY2021029), Key programs of the Suzhou Vocational Health College (Grant No. szwzy202102), and Qing-Lan Project of Jiangsu Province in China (2021).

## Conflict of Interest

The authors declare that the research was conducted in the absence of any commercial or financial relationships that could be construed as a potential conflict of interest.

## Publisher’s Note

All claims expressed in this article are solely those of the authors and do not necessarily represent those of their affiliated organizations, or those of the publisher, the editors and the reviewers. Any product that may be evaluated in this article, or claim that may be made by its manufacturer, is not guaranteed or endorsed by the publisher.

## Supplementary Material

Supplementary Figure 1 The definition of radiosensitivity and radioresistant. **(A)** Definition of radiosensitivity. **(B)** Definition of non-radiosensitivity. RR, radioresistant; NRT, non-radiotherapy; RS, radiosensitivity; RT, radiotherapy.

Supplementary Figure 2 The clustering tree of samples. **(A)** The clustering tree of outliers is not removed. **(B)** The clustering tree of samples without outliers.

Supplementary Figure 3 Forest plots of univariate Cox regression. **(A)** Forest plots of univariate Cox regression in TCGA (OS). **(B)** Forest plots of univariate Cox regression in TCGA (PFS). **(C)** Forest plots of univariate Cox regression in CGGA693. **(D)** Forest plots of univariate Cox regression in CGGA325. A, Astrocytoma; OA, Oligoastrocytoma; O, Oligodendroglioma.
