# Supplementary figures and images for: A Radiosensitivity Prediction Model Developed Based on Weighted Correlation Network Analysis of Hypoxia Genes for Lower-Grade Glioma
Source: Front Oncol. 2022 Feb 25;12:757686. doi: 10.3389/fonc.2022.757686 (PMC8916576; doi:10.3389/fonc.2022.757686)

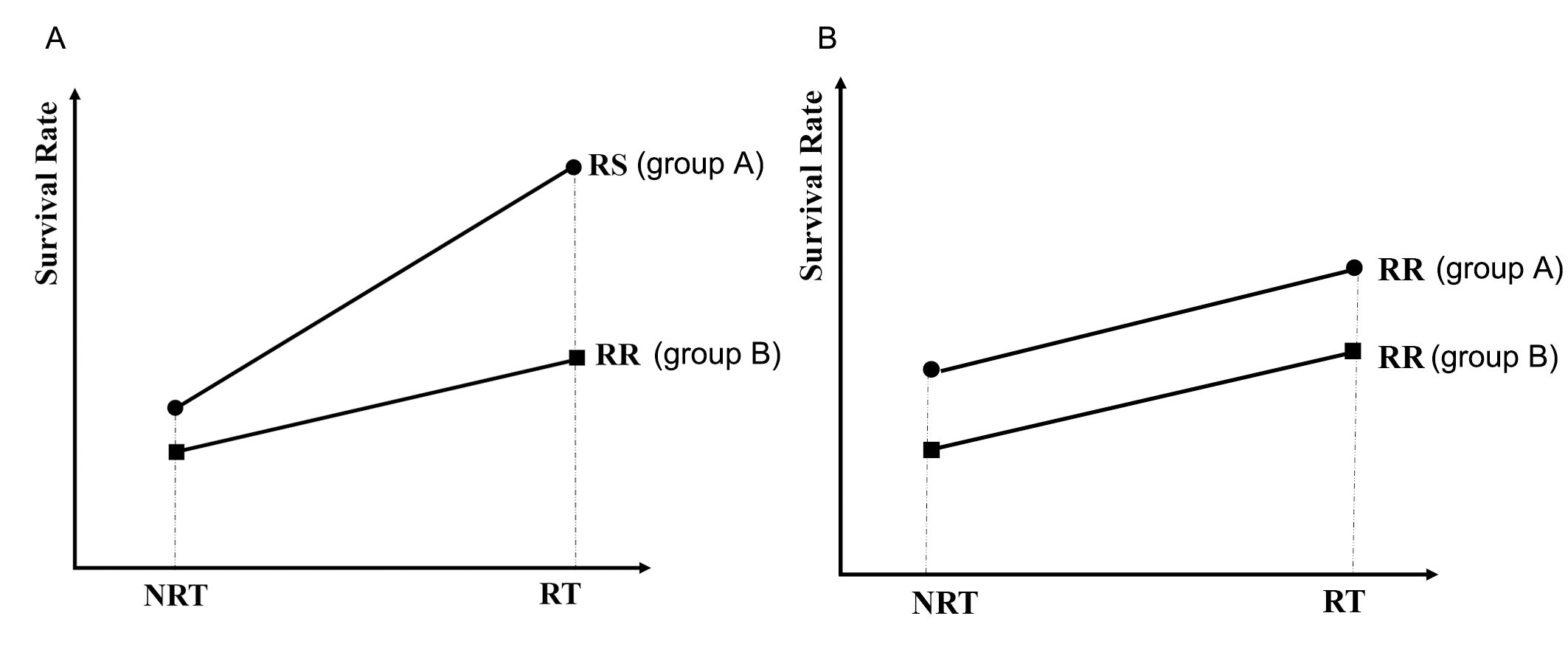

Supplement: Supplementary Figure 1 — The definition of radiosensitivity and radioresistant. (A) Definition of radiosensitivity. (B) Definition of non-radiosensitivity. RR, radioresistant; NRT, non-radiotherapy; RS, radiosensitivity; RT, radiotherapy. [file Image_1.jpeg]

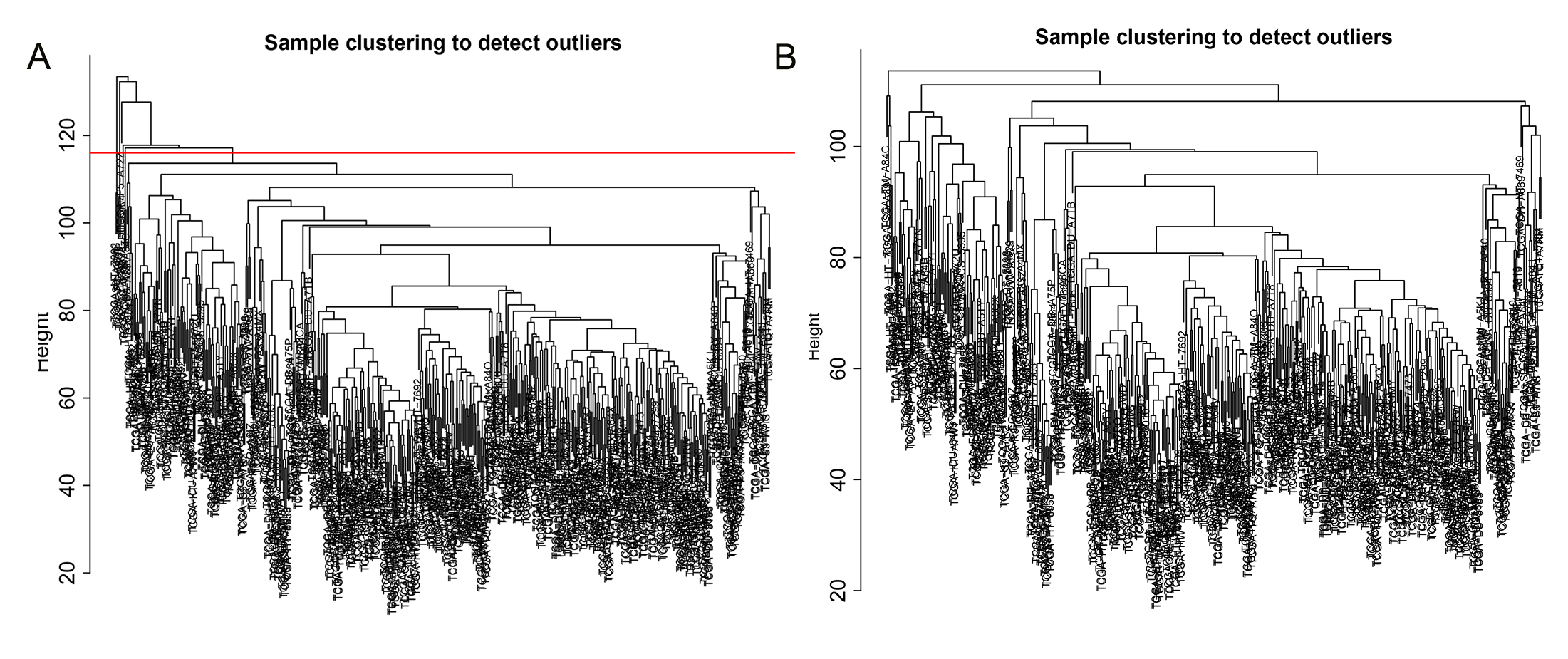

Supplement: Supplementary Figure 2 — The clustering tree of samples. (A) The clustering tree of outliers is not removed. (B) The clustering tree of samples without outliers. [file Image_2.jpeg]

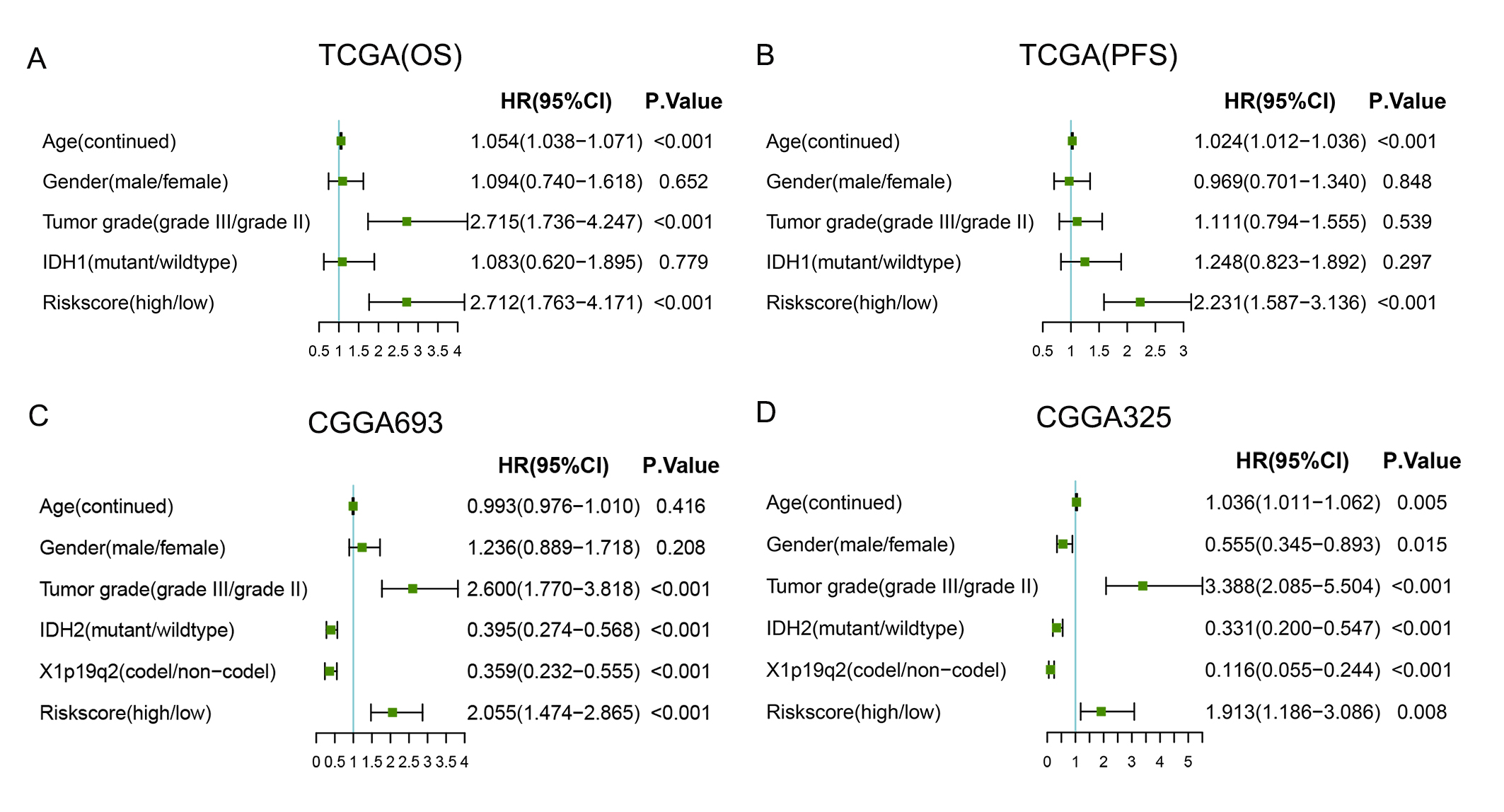

Supplement: Supplementary Figure 3 — Forest plots of univariate Cox regression. (A) Forest plots of univariate Cox regression in TCGA (OS). (B) Forest plots of univariate Cox regression in TCGA (PFS). (C) Forest plots of univariate Cox regression in CGGA693. (D) Forest plots of univariate Cox regression in CGGA325. A, Astrocytoma; OA, Oligoastrocytoma; O, Oligodendroglioma. [file Image_3.jpeg]
