# Supplementary material for: A Radiosensitivity Prediction Model Developed Based on Weighted Correlation Network Analysis of Hypoxia Genes for Lower-Grade Glioma
Source: Front Oncol. 2022 Feb 25;12:757686. doi: 10.3389/fonc.2022.757686 (PMC8916576; doi:10.3389/fonc.2022.757686)
Supplement: Supplementary file 4 [file Table_1.docx]

**Supplementary Materials**

**Supplemental Table 1 |** Basic patient characteristics in TCGA dataset

|  | **N** | **Median(range)/ratio** |
| --- | --- | --- |
| **Age** | 466 | 33(41-53) |
| **OS time (Months)** | 466 | 23.82(14.38-33.06) |
| **OS status** |  |  |
| Death | 119 | 25.54 |
| Censor | 347 | 74.46 |
| **PFS time (Months)** |  | 18.11(10.41-32.70) |
| **PFS status** |  |  |
| Death | 183 | 39.27 |
| Censor | 283 | 60.73 |
| **Gender** |  | 0.00 |
| Male | 255 | 54.72 |
| Female | 211 | 45.28 |
| **Laterality** |  | 0.00 |
| Left | 228 | 48.93 |
| Midline | 7 | 1.50 |
| Right | 227 | 48.71 |
| **Histology** |  | 0.00 |
| Astrocytoma | 172 | 36.91 |
| Oligoastrocytoma | 124 | 26.61 |
| Oligodendroglioma | 170 | 36.48 |
| **Tumor grade** | | Tumor grade |
| II | 223 | 47.85 |
| III | 243 | 52.15 |
| **Targeted molecular therapy** | | |
| Yes | 263 | 56.44 |
| No | 192 | 41.20 |
| **Radiotherapy** | | Radiotherapy |
| Yes | 318 | 68.24 |
| No | 148 | 31.76 |

**Supplemental Table 2 |** Basic patient characteristics in CGGA693 and CGGA325 datasets

|  |  | **CGGA693** | | | **CGGA325** | |
| --- | --- | --- | --- | --- | --- | --- |
|  | **N** | **Median(range)/ratio** | | | **N** | **Median(range)/ratio** |
| **Age** | 407 | 41(33-46.75) | | 168 | | 39(34.75-45) |
| **OS time** | 407 | 48.72(23.06-75.89) | | | 168 | 72.52(21.72-120.91) |
| **OS status** |  |  |  | |  |  |
| Death | 187 | 45.95 | | | 87 | 51.79 |
| Censor | 220 | 54.05 | | | 81 | 48.21 |
| **Gender** |  |  |  | |  |  |
| Male | 229 | 56.27 | | | 104 | 61.90 |
| Female | 178 | 43.73 | | | 64 | 38.10 |
| **Tumor grade** | |  |  | |  |  |
| II | 164 | 40.29 | | | 97 | 57.74 |
| III | 243 | 59.71 | | | 71 | 42.26 |
| **IDH status** |  |  |  | |  |  |
| Mutant | 280 | 68.80 | | | 125 | 74.40 |
| Wildtype | 90 | 22.11 | | | 43 | 25.60 |
| NA | 37 | 9.09 | | |  |  |
| **X1p19q** |  |  |  | |  |  |
| Codel | 122 | 29.98 | | | 55 | 32.74 |
| Non-codel | 249 | 61.18 | | | 112 | 66.67 |
| NA | 36 | 8.85 | | | 1 | 0.60 |
| **MGMTp** |  |  |  | |  |  |
| methylated | 197 | 48.40 | | | 83 | 49.40 |
| un-methylated | 125 | 30.71 | | | 69 | 41.07 |
| NA | 75 | 18.43 | | | 16 | 9.52 |
| **Radiotherapy** | |  |  | |  |  |
| Yes | 308 | 75.68 | | | 139 | 82.74 |
| No | 99 | 24.32 | | | 29 | 17.26 |
